# Skeletal and dental age estimation via postmortem computed tomography in Polish subadults group

**DOI:** 10.1007/s00414-023-03005-1

**Published:** 2023-05-01

**Authors:** Oleksiy Lopatin, Marta Barszcz, Krzysztof Jerzy Woźniak

**Affiliations:** 1 Chair and Department of Forensic Medicine, Jagiellonian University Medical College, Grzegórzecka 16, 31-531 Kraków, Poland; 2 Doctoral School of Medical and Health Sciences, Jagiellonian University Medical College, Kraków, Poland

**Keywords:** Age estimation, PMCT, Skeletal age estimation, Dental age estimation, Primary and secondary ossification centers, Deciduous teeth

## Abstract

This article is a retrospective analysis of postmortem computed tomography scans of ossification stages of the anterior and posterior intra-occipital sutures, the anterior arch of the atlas, and the neurocentral junction of the axis. We also analyzed the development of secondary ossification centers in the proximal humeral, femoral, and tibial epiphyses, and the distal femoral and tibial epiphyses. Additionally, the development of primary ossification centers in the wrist and metacarpals, and maxillary and mandibular deciduous tooth maturation. A total of 58 cadavers (35 males, 23 females), whose age ranged from 3rd month of pregnancy to 14 years, were analyzed. The results of this study show that analysis of synchondrosis closure, primary, and secondary ossification center development and deciduous tooth changes are a good tool for age estimation in subadults group (fetuses, newborns, infants, and children). The results of the study in a Polish population are consistent with those reported by other authors.

## Introduction

Age estimation is a complementary technique in cases involving establishing the identity of unidentified corpses [1]. Age estimation plays an important role in postmortem identification in archeological and forensic anthropology. Along with the sex and stature of the individual, his or her estimated age facilitates identification of human remains [1–3]. Researchers use anthropometric methods of age estimation in the form of measuring the length of bone shafts and observing the presence of primary and secondary ossification centers [4, 5]. Skeletal and dental age estimation in subadults (fetuses, newborns, infants, and children) [6] is still based on the methods involving assessment of human remains [7–9]. Ultrasonography and radiological methods, such as x-rays and computed tomography, are used in order to better assess bone ossification and tooth formation [10–12]. Earlier relevant studies in the Polish population were based on anthropometric measurements of bone shafts and primary ossification centers in the skull [13–17].

The purpose of this study was to estimate the skeletal and dental age in a Polish population aged between 3rd prenatal month and 14 years via analyzing the process of synchondrosis ossification, the emergence of primary and secondary ossification centers, and the maturation and loss of deciduous teeth. Our age estimation results were compared with those reported by other authors. The results of ossification analysis were stratified by the individual stages of bone fusion and formation of selected ossification centers, which were compared against the chronological age, with respect to pre-defined periods of skeletal maturity [18]. Another purpose of the study was to compare the process of development in a high number of primary and secondary ossification centers in bones and deciduous teeth searching for any similarities in the time of skeletal and dental maturation that could be useful in determining the age of children in the present-day Polish population. The results of our analysis may be equally useful both in establishing the identity of the deceased and in the identification of the living.

## Material and methods

This study was a retrospective analysis of postmortem computed tomography (PMCT) images depicting ossification stages of the anterior and posterior intra-occipital sutures, the anterior arch of the atlas, and the neurocentral junction of the axis. PMCT images were also analyzed for the development of secondary ossification centers at the proximal humeral, femoral, and tibial epiphyses and the distal femoral and tibial epiphyses. Moreover, the development of primary ossification centers in the wrist and midfoot was assessed. PMCT images were also analyzed to assess maxillary and mandibular deciduous tooth maturation. The stages of ossification were analyzed with reference to the periods of skeletal maturation defined in the Digital Atlas of Skeletal Maturity by Gilsanz and Ratib: infancy (males: birth to 14 months of age; females: birth to 10 months of age); toddlers (males: 14 months to 3 years of age; females: 10 months to 2 years of age); pre-puberty (males: 3 years to 9 years of age; females: 2 years to 7 years of age); early and mid-puberty (males: 9 years to 14 years of age; females: 7 years to 13 years of age) [18].

A total of 58 cadavers (35 males and 23 females) aged between 3^rd^ prenatal month and 14 years were assessed. The PMCT scans were conducted at the Forensic Medicine Department of Jagiellonian University Medical College in the period 2012–2016. The equipment used was a Siemens Somatom Emotion 16 computed tomography scanner, 130 kV, 240 mAs, collimation 16 × 0.6, pitch 0.85, slice thickness of 0.75 mm for the head, and 1.5 mm for the torso and limbs. Image reconstruction was obtained via OsiriX software v.5.5.1, Pixmeo SARL, Switzerland.

Statistical analyses were conducted with IBM SPSS Statistics 28 software.

Spearman’s Rho test was used to check, if linear connection between changes in ossification center and age occur. The level of statistical significance was adopted at *p*=0.001. Descriptive statistics were used to analyze dentition-related data.

In this paper the individual teeth are referred to by their two-digit notations, according to the FDI World Dental Federation system.

The Wilcoxon signed-rank test was used to assess whether the methods of assessing the changes in ossification centers and tooth maturity were justifiable.

**Fig. 1:**
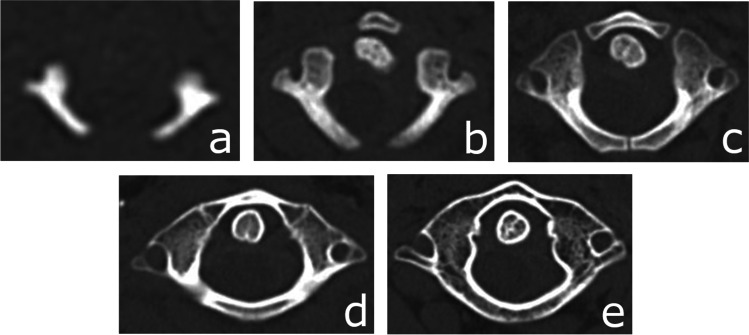
Computed tomography images depicting consecutive stages of ossification in the anterior arch of the atlas: **A** Stage 1 – no primary ossification center in the anterior arch of the atlas; **B** Stage 2 – the ossification center of the anterior arch of the atlas is present, its shape is irregular; **C** Stage 3 – a mature form of the anterior arch of the atlas, with open synchondroses; **D** Stage 4 – the anterior synchondroses are partially closed; **E** Stage 5 – complete closure of the anterior synchondroses

**Fig. 2:**
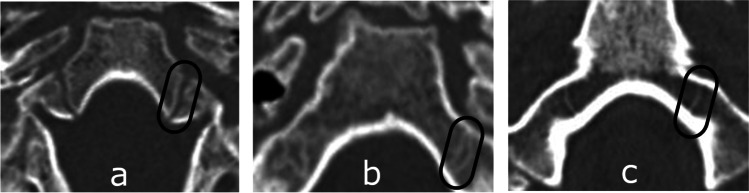
Computed tomography images depicting consecutive stages of anterior intra-occipital suture ossification: **A** Stage 1 – no ossification; **B** Stage 2 – partial ossification; **C** Stage 3 – complete ossification

**Fig. 3:**
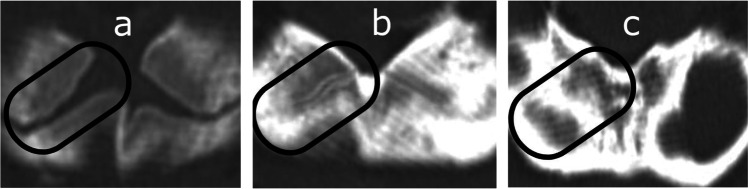
Computed1 tomography images depicting consecutive stages of posterior intra-occipital suture ossification: **A** Stage 1 – no ossification; **B** Stage 2 – partial ossification; **C** Stage 3 – complete ossification

**Fig. 4:**
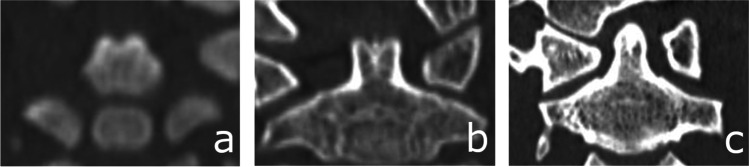
Computed tomography images depicting consecutive stages of ossification in the neurocentral junction of the axis: **A** Stage 1 – no ossification; **B** Stage 2 – partial ossification; **C** Stage 3 – complete ossification

**Fig. 5:**
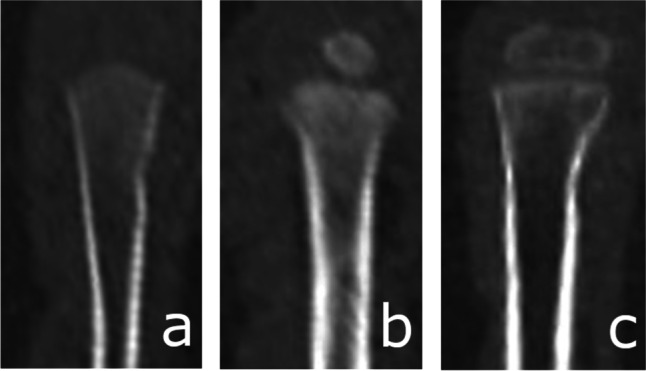
Computed tomography images depicting the development of secondary ossification center in the proximal humeral epiphysis: **A** Stage 1 – no secondary ossification centers; **B** Stage 2 – the presence of an irregularly shaped secondary center of ossification that occupies less than half of the width of the metaphysis; **C** Stage 3 – development of ossified epiphysis, initially the epiphyseal plate spans more than half of the width of the metaphysis, and towards the end of development the epiphysis and the metaphysis are of comparable width

**Fig. 6:**
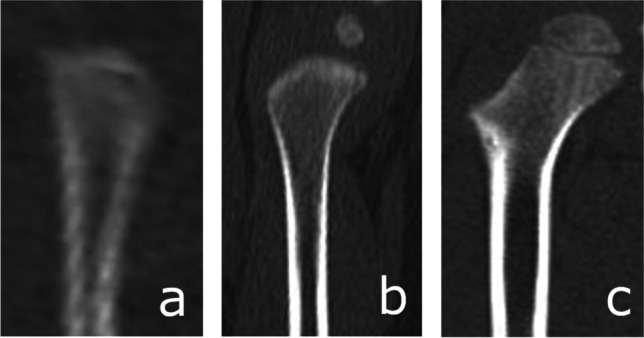
Computed tomography images depicting the development of secondary ossification center in the proximal femoral epiphysis: **A** Stage 1 – no secondary ossification centers; **B** Stage 2 – the presence of an irregularly shaped secondary center of ossification that occupies less than half of the width of the metaphysis; **C** Stage 3 – development of ossified epiphysis, initially the epiphyseal plate spans more than half of the width of the metaphysis, and towards the end of development the epiphysis and the metaphysis are of comparable width

**Fig. 7:**
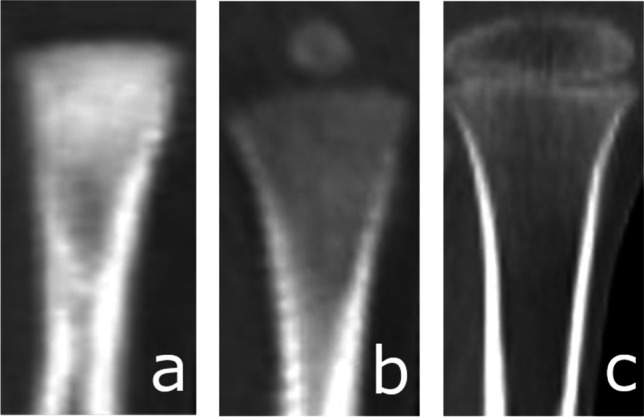
Computed tomography images depicting the development of secondary ossification center in the proximal tibial epiphysis: **A** Stage 1 – no secondary ossification centers; **B** Stage 2 – the presence of an irregularly shaped secondary center of ossification that occupies less than half of the width of the metaphysis; **C** Stage 3 – development of ossified epiphysis, initially the epiphyseal plate spans more than half of the width of the metaphysis, and towards the end of development the epiphysis and the metaphysis are of comparable width

**Fig. 8:**
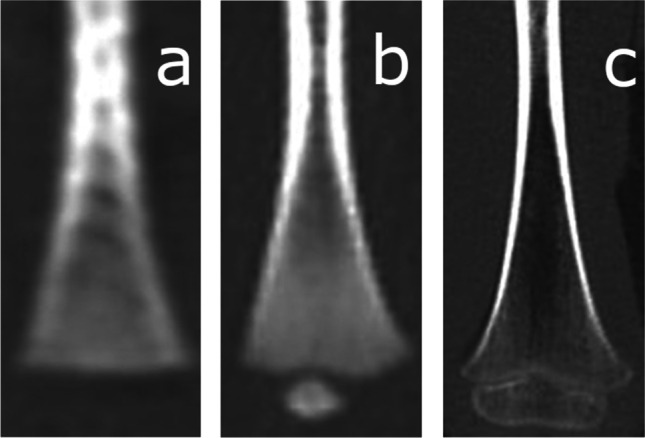
Computed tomography images depicting the development of secondary ossification center in the distal femoral epiphysis: **A** Stage 1 – no secondary ossification centers; **B** Stage 2 – the presence of an irregularly shaped secondary center of ossification that occupies less than half of the width of the metaphysis; **C** Stage 3 – development of ossified epiphysis, initially the epiphyseal plate spans more than half of the width of the metaphysis, and towards the end of development the epiphysis and the metaphysis are of comparable width

**Fig. 9:**
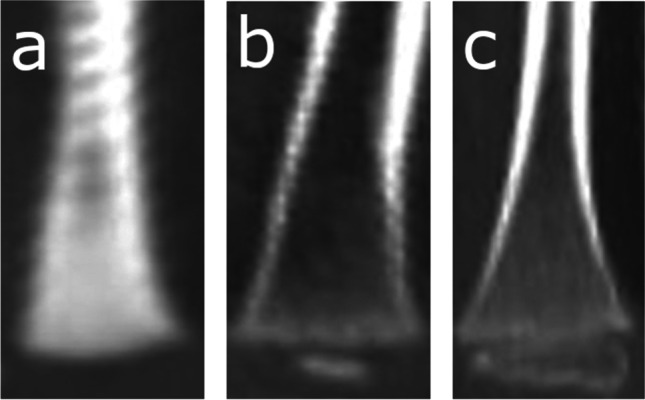
Computed tomography images depicting the development of secondary ossification center in the distal tibial epiphysis: **A** Stage 1 – no secondary ossification centers; **B** Stage 2 – the presence of an irregularly shaped secondary center of ossification that occupies less than half of the width of the metaphysis; **C** Stage 3 – development of ossified epiphysis, initially the epiphyseal plate spans more than half of the width of the metaphysis, and towards the end of development the epiphysis and the metaphysis are of comparable width

**Fig. 10:**
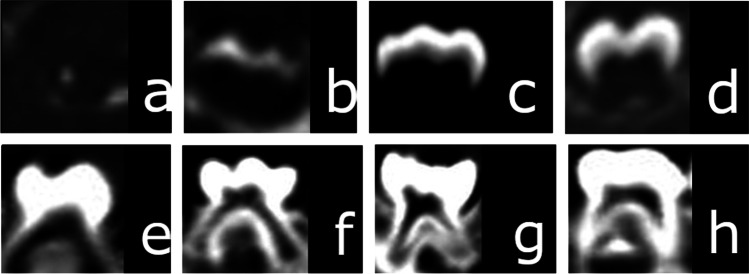
Computed tomography scans showing deciduous tooth maturation: **A** Stage 1 – beginning of calcification, calcification of single occlusal points. **B** Stage 2 – calcification points fuse to form the occlusal surface. **C** Stage 3 – dentin is visible. **D** Stage 4 – beginning of root formation, a dentine spicule. **E** Stage 5 – root length remains shorter than crown height. **F** Stage 6 – full root length has not been reached, canals are divergent in the apical area. **G** Stage 7 – root length is nearly full, apical canal walls are parallel or slightly convergent. **H** Stage 8 – completely mature root

The process of ossification of the anterior arch of the atlas [Fig. 1] was assessed and classified according to a five-stage scale. Ossification of the anterior [Fig. 2] and posterior [Fig. 3] intra-occipital suture and neurocentral junction of the axis [Fig. 4] and the development of secondary ossification centers in the proximal humeral [Fig. 5], femoral [Fig. 6], and tibial [Fig. 7] epiphyses and distal femoral [Fig. 8] and tibial [Fig. 9] epiphyses, and the development of primary ossification centers in wrist and midfoot bones were assessed and classified according to a three-stage scale. The development of maxillary and mandibular deciduous teeth was assessed with the Demirjian’s method [19] [Fig. 10]. We modified the Demirjian’s system to include an additional stage (stage 9), to mark the stage, when the deciduous tooth is no longer present in the dental alveolus.

## **Result**s

### Statistical analysis of ossification centers

Statistical analysis suggests progressive ossification at all ossification centers with age. The level of significance adopted for all conducted correlation analyses was *p*<0.001. The Spearman’s correlation coefficient (Rho) was positive in all analyses, which indicates that ossification centers maturation progresses with age.

Analysis of data from all study subjects showed the strongest relationship between the two variables (age and the stage of epiphyseal maturation) at Prox Fem (*p*<0.001; Rho=0.89). This finding shows a very strong correlation, when interpreted according to Stanisz scale. Similar strength of correlation can be also seen in the following ossification centers Ant Arch, Prox Hum, Prox Tib, Dist Tib, and Wrist Prim. Somewhat weaker though still relatively strong correlations were observed at the following ossification centers Ant Synch, Post Synch, Neur Axis, and Midfoot Prim [Tables 1, 2, 3, 4].

**Table 1:** Ossification of the anterior arch of the atlas; indication of sex: ♂-males, without the symbol-females

No primary ossification centers Stage 1 Min–max age		Primary ossification center present Stage 2 Min–max age		A mature form with open S Stage 3 Min–max age		Partially closed S Stage 4 Min–max age		Complete S closure Stage 5 Min–max age	
4pm♂	13mos♂	2mos♂	3yo♂	19mos♂	7yo♂	9yo♂	11yo♂	7yo♂	14yo♂
nb	6mos	nb	6mos	11mos	5yo	4yo	7yo	8yo	14yo

**Table 2:** Stages of synchondrosis ossification of anterior intra-occipital synchondrosis, posterior intra-occipital synchondrosis and neurocentral junction of axis; indication of sex: ♂-males, without the symbol-females

Ossification centers	No ossification Stage 1 Min–max age		Partial ossification Stage 2 Min–max age		Complete ossification Stage 3 Min–max age	
Ant Synch	4pm♂	3yo♂	7yo♂	10yo♂	7yo♂	14yo♂
	nb	3yo	4yo	8yo	8yo	14yo
Post Synch	4pm♂	9mos♂	2mos♂	19mos♂	3yo♂	14yo♂
	nb	3mos	6mos	3yo	2yo	14yo
Neur Axis	4pm♂	14mos♂	19mos♂	11yo♂	7yo♂	14yo♂
	nb	3yo	3yo	9yo	5yo	14yo

**Table 3:** Secondary centers of ossification of proximal humeral secondary ossification centers, proximal femoral secondary ossification centers, distal femoral secondary ossification centers, proximal tibial secondary ossification centers and distal tibial second*;* indication of sex: ♂-males, without the symbol-females

Secondary ossification centers	No secondary ossification centers Stage 1 Min–max age		Secondary ossification centers present Stage 2 Min–max age		Epiphyseal maturation Stage 3 Min–max age	
Prox Hum	3pm♂	3mos♂	nb♂	9mos♂	13mos♂	9yo♂
	nb	nb	nb	11mos	2yo	9yo
Prox Fem	3pm♂	4mos♂	4mos♂	9mos♂	13mos♂	10yo♂
	nb	3mos	6mos	6mos	11mos	9yo
Dist Fem	3pm♂	3mos♂	9pm♂	4mos♂	6mos♂	9yo♂
	1mo	1mo	nb	6mos	3mos	6yo
Prox Tib	3pm♂	3mos♂	9pm♂	4mos♂	9mos♂	9yo♂
	nb	1mo	nb	6mos	3mos	4yo
Dist Tib	3pm♂	4mos♂	4mos♂	14mos♂	13mos♂	9yo♂
	nb	3mos	6mos	6mos	11mos	8yo

**Table 4:** Development of primary ossification centers in the wrist and midfoot; indication of sex: ♂-males, without the symbol-females

Wrist and Midfoot primary ossification centers	No primary ossification centers Stage 1 Min–max age		Primary ossification center development Stage 2 Min–max age		All primary ossification centers are present Stage 3 Min–max age	
Wrist Prim	3pm♂	4mos♂	4mos♂	10yo♂	10yo♂	14yo♂
	nb	nb	3mos	8yo	8yo	14yo
Midfoot Prim	3pm♂	4mos♂	nb♂	14mos♂	19mos♂	11yo♂
	nb	nb	nb	2yo	2yo	8yo

Spearman’s Rho test was used to test for potential associations between the subject’s sex and the relationship between age and ossification center maturation. The strongest relationship between age and ossification center maturation was observed at Dist Tib (*p*<0.001; Rho=0.91) and Prox Fem (*p*<0.001; Rho=0.90) in females and at Prox Hum (*p*<0.001; Rho=0.86) in males. Similar levels of this relationship were also observed at Ant Synch, Ant Arch, and Wrist Prim in females and at Prox Fem, Dist Fem, and Dist Tib in males. The weakest though still relatively strong correlation between the analyzed variables (age and ossification center maturation) was found at Post Synch (*p*<0.001; Rho=0.69) and at Ant Synch (*p*<0.001; Rho=0.63), Neur Axis (*p*<0.001; Rho=0.64), Ant Arch (*p*<0.001; Rho=0.67), and Midfoot Prim (*p*<0.001; Rho=065) in males. High degrees of correlation were observed at Ant Synch, Neur Axis, Ant Arch, Prox Hum, Dist Fem, Prox Tib, Wrist Prim, and Midfoot Prim in women, and Prox Hum, Prox Fem, Prox Tib, Wrist Prim, and Dist Tib in men [Tables 1–4].

In males, the absence of primary ossification centers, in the wrist and midfoot, the absence of secondary ossification centers in the proximal humeral epiphysis, proximal and distal femoral epiphyses, and proximal and distal tibial epiphyses, and the absence of fusion in the posterior intra-occipital suture are not observed after infancy (males: birth–14mos). The presence of all primary ossification centers in the midfoot is observed in males after infancy (males: birth–14mos). Partial ossification of the posterior intra-occipital suture is no longer observed in males after the toddler period (males: 14mos–3yo). The stage of complete ossification of the anterior intra-occipital suture and neurocentral junction of the axis is observed in males after the toddler period (males: 14mos–3yo). The stage of epiphyseal maturation in the proximal humeral epiphysis, proximal and distal femoral epiphyses, and proximal and distal tibial epiphyses are no longer observed in males after pre-puberty (males: 3–9yo). Conversely, the stage when all wrist bones are present and the stage of deciduous tooth loss are observed in the male group after pre-puberty (males: 3–9yo) [Tables 5, 7].

**Table 5:** The number of males at the individual stages of bone maturation process and selected ossification center formation stratified by their chronological age based on periods of skeletal maturity

Ossification centers	chronological age	Stage 1 No ossification	Stage 2 Partial ossification	Stage 3 Complete ossification
Ant Synch	<14mos	22	0	0
	14mos–3yo	4	0	0
	4–9yo	0	1	2
	10–14yo	0	1	5
Post Synch	<14mos	20	2	0
	14mos–3yo	0	2	2
	4–9yo	0	0	3
	10–14yo	0	0	6
Neur Axis	<14mos	22	0	0
	14mos–3yo	1	3	0
	4–9yo	0	0	3
	10–14yo	0	1	5
Secondary ossification centers	Chronological age	Stage 1 No secondary ossification centers	Stage 2 Secondary ossification centers present	Stage 3 Epiphyseal maturation
Prox Hum	<14mos	15	6	1
	14mos–3yo	0	0	4
	4–9yo	0	0	3
	10–14yo	0	0	0
Prox Fem	<14mos	19	2	1
	14mos–3yo	0	0	4
	4–9yo	0	0	3
	10–14yo	0	0	1
Dist Fem	<14mos	8	12	2
	14mos–3yo	0	0	4
	4–9yo	0	0	3
	10–14yo	0	0	0
Prox Tib	<14mos	12	8	2
	14mos–3yo	0	0	4
	4–9yo	0	0	3
	10–14yo	0	0	0
Dist Tib	<14mos	19	2	1
	14mos–3yo	0	1	3
	4–9yo	0	0	3
	10–14yo	0	0	0
Primary ossification centers	Chronological age	Stage 1 No primary ossification centers	Stage 2 Primary ossification center development	Stage 3 All primary ossification centers are present
Wrist Prim	<14mos	19	3	0
	14mos–3yo	0	4	0
	4–9yo	0	3	0
	10–14yo	0	1	5
Midfoot Prim	<14mos	13	9	0
	14mos–3yo	0	1	3
	4–9yo	0	0	3
	10–14yo	0	0	3

In females, the lack of posterior intra-occipital suture ossification, absent secondary centers of ossification in the proximal humeral epiphysis, proximal and distal femoral epiphyses, proximal and distal tibial epiphyses, absent wrist and midfoot bones are no longer observed after the end of the infancy period (females: birth–10mos). Partial ossification of the anterior intra-occipital suture and neurocentral junction of the axis is observed after the toddler period (females: 10mos–2yo). Complete ossification of the anterior intra-occipital suture and the presence of all wrist bones are observed and the process of distal femoral and proximal tibial epiphyseal maturation is no longer observed in females after the pre-puberty period (females: 2–7yo). After this period, deciduous tooth loss occurs [Tables 6, 7].

**Table 6:** The number of females at the individual stages of bone fusion and selected ossification center formation stratified by their chronological age based on periods of skeletal maturity

Ossification centers	Chronological age	Stage 1 No ossification	Stage 2 Partial ossification	Stage 3 Complete ossification
Ant Synch	<10mos	7	0	0
	10mos–2yo	3	0	0
	3–7yo	2	5	0
	8–13yo	0	1	5
Post Synch	<10mos	5	2	0
	10mos–2yo	0	2	1
	3yo–7yo	0	3	4
	8–13yo	0	0	6
Neur Axis	<10mos	7	0	0
	10mos–2yo	3	0	0
	3–7yo	1	3	3
	8–13yo	0	1	5
Secondary ossification centers	Chronological age	Stage 1 No secondary ossification centers	Stage 2 Secondary ossification centers present	Stage 3 Epiphyseal maturation
Prox Hum	<10mos	1	6	0
	10mos–2yo	0	1	2
	3–7yo	0	0	7
	8–13yo	0	0	2
Prox Fem	<10mos	5	2	0
	10mos–2yo	0	0	3
	3–7yo	0	0	6
	8–13yo	0	0	1
Dist Fem	<10mos	1	4	2
	10mos–2yo	0	0	3
	3–7yo	0	0	5
	8–13yo	0	0	0
Prox Tib	<10mos	2	3	2
	10mos–2yo	0	0	3
	3–7yo	0	0	4
	8–13yo	0	0	0
Dist Tib	<10mos	5	2	0
	10mos–2yo	0	0	3
	3–7yo	0	0	3
	8–13yo	0	0	2
Primary ossification centers	chronological age	Stage 1 No primary ossification centers	Stage 2 Primary ossification center development	Stage 3 All primary ossification centers are present
Wrist Prim	<10mos	3	4	0
	10mos–2yo	0	3	0
	3–7yo	0	7	0
	8–13yo	0	1	5
Midfoot Prim	<10mos	1	6	0
	10mos–2yo	0	2	1
	3–7yo	0	0	5
	8–13yo	0	0	2

**Table 7:** Maturation of deciduous teeth; indication of sex: ♂- males, without the symbol- females

Deciduous tooth numbers	Stage 1 (stages 1–7) Min–max age		Stage 2 (stage 8) Min–max age		Tooth loss Stage 3 (stage 9) Min–max age	
51/61/71/81	nb♂	19mos♂	19mos♂	10yo♂	9yo♂	14yo♂
	nb	2yo	2yo	7yo	8yo	14yo
52/62/72/82	nb♂	19mos♂	19mos♂	10yo♂	9yo♂	14yo♂
	nb	2yo	2yo	7yo	8yo	14yo
53/63/73/83	nb♂	3yo♂	3yo♂	13yo♂	13yo♂	14yo♂
	nb	3yo	5yo	11yo	11yo	12yo
54/64/74/84	nb♂	3yo♂	3yo♂	11yo♂	13yo♂	14yo♂
	nb	2yo	2yo	11yo	8yo	12yo
55/65/75/85	3mos♂	3yo♂	3yo♂	11yo♂	13yo♂	14yo♂
	nb	3yo	5yo	9yo	11yo	14yo

The stage of absent ossification of the posterior intra-occipital suture, absent secondary ossification centers in the proximal humeral epiphysis, proximal and distal femoral epiphyses, proximal and distal tibial epiphyses, and absent wrist and midfoot bone ossification are no longer observed in either sex after the infancy period is over (males: birth–14mos, females: birth–10mos) [Tables 5, 6].

### Statistical analysis of teeth

Tooth maturation (stages 1–7) in the case of deciduous maxillary first molars in males takes longer than in females. The minimum age of tooth maturity (stage 8) for deciduous mandibular incisors (teeth 71, 81, 72, and 82) in males was 1.5 years. The maximum age at which the tooth maturity stage was observed was 10 years for deciduous central and lateral maxillary and mandibular incisors in males and 13 years for deciduous canines in males. The beginning of tooth loss (stage 9) is observed decidedly later than the beginning of tooth maturity (stage 8). Tooth loss (stage 9) can be observed from the age of 8 years, starting with the loss of deciduous mandibular central incisors, all lateral incisors and of the mandibular right first molar in female. Tooth maturity (stage 8) in females is shorter than in males in the case of all deciduous teeth with the exception of the maxillary and mandibular first molars, in which the tooth maturity stage is shorter in males. The loss of deciduous teeth is observed in both sexes after pre-puberty (males: 3–9yo; females: 2–7yo) (Table 7).

## Intraobserver reliability

Additionally, we decided to verify the reliability of our study measurements, by re-analyzing ossification center maturation. The ossification center and tooth maturation assessment results from the 34 re-analyzed cases showed a *p*-value that indicated the lack of statistical significance (*p*>0.05).

## Discussion

In our study, we analyzed the appearance of primary and secondary ossification centers in the skeleton and deciduous tooth development over time. These age estimation methods are also recommended by Cunha et al. in this age group [6]. Other authors [10, 20] emphasize the importance of analyzing primary and secondary ossification centers simultaneously. Most studies in this age group were based on radiographic assessments in living, hospitalized individuals. Postmortem imaging may become increasingly important because the application of clinical radiology modalities is not recommended for individuals under 18 years of age [21, 22]. Thus, our study shows potential opportunity of the use of postmortem computed tomography in age estimation. The comparison of the results of our study with those by other authors revealed some similarities.

No ossification centers present in the anterior arch of the atlas was most common in patients younger than 12 months in Junewick study et al. [23]. In our study, the stage of absent ossification in the anterior arch of the atlas is no longer observed after 14 months of age in male groups and after 10 months of age in female group. Complete ossification of the anterior arch of the atlas was reported by Karwacki et al. [11] in children aged approximately 12 years, and is generally observed between 4.5 and 17 years of age. Wu et al. [24] noticed that complete closed Ant Arch synchondrosis observed at a median age of 8 years in males and 6.3 years in females. In our study, the minimum age of complete synchondrosis ossification was 7 years in males and 8 years in females. These findings fall within the age ranges reported by Karwacki et al. [11] and are similar to that observed by Wu et al. [24].

Lottering study et al. [25] showed strong relationship between age and active-complete stage at the following ossification centers Ant Synch (*p*<0.001) and Post Synch (*p*<0.10). In present, study showed the level of significance (*p*<0.001) in the Ant Synch and Post Synch ossification centers in all stages, which indicates that ossification centers maturation progresses with age. Cardoso study et al. [26] showed that fusion occurs first in the Post Synch 1 years–5 years and last in the Ant Synch between 3 and 7 years of age, with no significant sex differences. In our study complete ossification observed the same first in Post Synch in males group at the age of 3 years and in females group at the age of 2 years. Ant Synch finished ossification noticed in males group at the age 7 years and in the females group at the age of 8 years.

Karwacki et al. [11] reported a completely ossified neurocentral junction of the axis in 80% of children aged 7–9 years, with the earliest case observed at the age of 6.5 years. In our study the minimum age of complete ossification in the neurocentral junction of the axis in males was 7 years, which is similar to that observed by Karwacki et al. [11].

Studies by Ogden et al. [27] demonstrated the appearance of a secondary center of ossification in the proximal humeral epiphysis at the age of 2–3 months. Kwong et al. [28] noticed appearance of Prox Hum at the age of 4 months. This time window correlates with that observed in our study. Pryse-Davies et al. [29] reported the beginning of ossification in the proximal humeral epiphysis at 37 weeks of gestation, in the distal femoral epiphysis at 29 weeks of gestation, and in the proximal tibial epiphysis at 37 weeks of gestation. Mahony et al. [30] noticed that the Dist Fem was first seen at 29 gestation weeks. Our study showed the secondary centers of ossification in the distal femoral and proximal tibial epiphyses emerging during the prenatal period. Schaefer et al. [5] reported the appearance of the secondary center of ossification in the head of the femur during the first year of life and a secondary center of ossification in the distal tibial epiphysis at the age of 3–10 months. Parvaresh et al. [31] reported the appearance of Prox Fem before second year of life in either sex. These observations are consistent with those of our study for proximal femoral and distal tibial epiphyseal ossification.

According to Gilsanz et al. [18] the last primary ossification center to appear in the wrist was in the pisiform bone during Early and Mid-puberty (males: 9–14y; females: 7–13y). In our study, all primary centers of ossification in the wrist were also present in both sexes during Early and Mid-puberty. Schaefer et al. [5] reported that the last primary ossification center in the midfoot appears in males between the age 4 and 5 years and in females between 2 and 3 years. In our study all bones of the midfoot were observed at the minimum age of 19 months in males and 2 years in females.

Liversidge et al. [32] reported the earliest observations of fully mature deciduous central incisors to be before the age of 2 years, lateral incisors – slightly over 2 years, canines – slightly over 3 years, first molars – under 2.5 years, and second molars – at approximately 3 years of age. Our study showed that the minimum age ranges for mature deciduous canines and second molars are similar to the corresponding ones reported by Liversidge et al. [32] in the male group.

The differences between the results of our study and those by other authors are presented in Table 8.

**Table 8:** Comparison of our findings with those of other radiographic studies; ♂- males, ♀- females

Authors Ossification centers	Imaging types Group ages Corpses	Age period with max. number of children in no ossification process	Age period with max. number of children in ossification process	Age period with max. number of children in finished ossification process
Junewick et.al. [23] Ant Arch	CT 0–96mos (0–8yo) *n*=871	0–12mos 0–1yo	37–48mos 3–4yo	85–96mos 7–8yo
Karwacki et.al. [11] Ant Arch	CT 0–17yo *n*=550	–	–	approximately 12yo
Wu et.al. [24] Ant Arch	CT 0–16yo *n*=910	–	–	♂median age 8yo ♀median age 6.3yo
Present study Ant Arch	CT corpses 3pm–14yo *n*=58	<14mos	14mos–3yo	♂ 10yo–14yo ♀ 8yo–13yo
Lottering et.al. [25] Ant Synch	CT 0–10yo *n*=585	♂ 0–2yo ♀ 0–2yo	♂ 2–5yo ♀ 2–5yo	♂ 6–8.5yo ♀ 6–8.5yo
Present study Ant Synch	CT corpses 3pm–14yo *n*=58	♂ <14mos ♀ <10mos	♂ 4–9yo ♀ 3–7yo	♂ 10–14yo ♀ 8–13yo
Lottering et.al. [25] Post Synch	CT 0–10yo *n*=585	♂ 0–1yo ♀ 0–1yo	♂ 1–2yo ♀ 0–2yo	♂ 3–6yo ♀ 3–7yo
Present study Post Synch	CT corpses 3pm–14yo *n*=58	♂ <14mos ♀ <10mos	♂ 0–3yo ♀ 0–2yo	♂ 10–14yo ♀ 8–13yo
Karwacki et.al. [11] Neur Axis	CT 0–17yo *n*=550	–	–	approximately 9yo
Present study Neur Axis	CT corpses 3pm–14yo *n*=58	♂ <14mos ♀ <10mos	♂ 14mos–3yo ♀ 3–7yo	♂ 10–14yo ♀ 8–13yo
Ogden et.al. [27] Prox Hum	x-ray corpses 9pm–14yo *n*=23	♂and ♀ <2mos	♂and ♀ 2mos–5yo	♂and ♀ 5–10yo
Pryse-Davies et.al. [29] Prox Hum	x-ray corpses 21gw–42gw *n*=164	♂and ♀ <37gw	♂and ♀41–42gw	–
Kwong et.al. [28] Prox Hum	MRI 2mos–17yo *n*=94	♂and ♀ <4mos	♂and ♀4mos–3yo	♂and ♀ 5–9yo
Present study Prox Hum	CT corpses 3pm–14yo *n*=58	♂and ♀ <14mos	♂and ♀ <10m–2yo	♂and ♀ 3–9yo
Parvaresh et.al. [31] Prox Fem	CT 2–32yo *n*=496	♂ <2yo ♀ <2yo	-	♂ <13yo ♀ <11yo
Present study Prox Fem	CT corp 3pm–14yo *n*=58	♂ <14mos ♀ <10mos	♂ <14mos ♀ <10mos	♂ 14mos-3yo ♀ 3–7yo
Mahony et.al. [30] Dist Fem	USG intrauterine >28gw *n*=116	♂and ♀ <34gw	♂and ♀ >35gw	–
Present study Dist Fem	CT corpses 3pm–14yo *n*=58	♂and ♀ <14mos	♂and ♀<14mos	♂and ♀ 14mos–7yo

The sample size in our study is smaller than those reported in other studies [11, 23–25, 31]. This small sample size is related to the scarcity of cases undergoing postmortem examinations in this age group at our Forensic Medicine Department. We hope that future studies on this topic and our collaboration with other Centers will allow to expand the number of analyzed cases, both male and female, in this age group. The differences in the age ranges assessed in our study and those assessed in other studies may be due to different sample sizes and different age ranges of the study groups. Most age estimation studies are conducted in living individuals undergoing diagnostic assessments in hospital settings. Inpatient diagnostic investigations are usually conducted with the use of imaging studies such as computed tomography, magnetic resonance imaging, and ultrasound. We would like to emphasize that it is sometimes difficult to compare computed tomography images obtained postmortem with those obtained in the living. Postmortem computed tomography often uses higher doses of radiation and includes the entire body, which would be impossible to do in the living. This is the reason why there are so few studies assessing living children in sufficient detail to compare the results with those obtained postmortem [Table 8].

## Conclusions

The results of our study indicate that analyzing the process of synchondrosis ossification, the emergence of primary and secondary ossification centers, and the maturation and loss of deciduous teeth is a useful tool in age estimation in subadults group (fetuses, newborns, infants and children). Statistical analysis suggests progressive ossification at all ossification centers with age. The level of significance adopted for all conducted correlation analyses was *p*<0.001. The Spearman’s correlation coefficient (Rho) was positive in all analyses, which indicates that ossification centers maturation progresses with age.

Most of the study in this age group based on radiographic examination on living people group in hospital environment. Our study shows positive trends on postmortem computed tomography in age estimation process.

The observations made in the Polish population are similar to those obtained in other studies. These similarities may be helpful in age estimation, which is part of individual identification.

The beginning of the stage of deciduous tooth loss considered in combination with the stages of selected skeletal centers of ossification may be another component in obtaining a more accurate age estimate.

Moreover, we demonstrated the value of assessing several ossification centers simultaneously, which is due to a partial temporal overlap in the stages of ossification.

At the same time, the results of our study showed consistent time windows for the individual periods of skeletal maturity in male and female subadults. Additionally, the strongest relationship between age and ossification center maturation was observed at Dist Tib (*p*<0.001; Rho=0.91) and Prox Fem (*p*<0.001; Rho=0.90) in females and at Prox Hum (*p*<0.001; Rho=0.90) in males. Nonetheless, further studies conducted in larger populations are needed.

## Abbreviations

*Ant Arch*: anterior arch of the atlas
*Ant Synch*: anterior intra-occipital synchondrosis
*Dist Fem*: distal femoral secondary ossification centers
*Dist Tib*: distal tibial secondary ossification centers
*Midfoot Prim*: midfoot primary ossification centers
*Neur Axis*: neurocentral junction of axis
*Prox Fem*: proximal femoral secondary ossification centers
*Prox Hum*: proximal humeral secondary ossification centers
*Post Synch*: posterior intra-occipital synchondrosis
*Prox Tib*: proximal tibial secondary ossification centers
*Wrist Prim*: wrist primary ossification centers
*S*: synchondrosis
*gw*: gestational weeks
*pm*: prenatal months
*nb*: newborn
*–*: non samples
